# Causes of Treatment Abandonment of Pediatric Cancer Patients – Experience in a Regional Cancer Centre in North East India

**DOI:** 10.31557/APJCP.2019.20.4.1133

**Published:** 2019

**Authors:** Munlima Hazarika, Rakesh Mishra, Bhargab Jyoti Saikia, Chidananda Bhuyan, C W Nyuthe, Anupam Sarma, Gaurav Kumar, Cliffton Sutnaga, Manoj Kalita, Partha Roy

**Affiliations:** 1 *Department of Medical Oncology,*; 2 *Department of Pathology,*; 3 *Population Based Cancer Registry, National Cancer Registry Programme (ICMR), Dr. B Borooah Cancer Institute, Gopinath Nagar, Guwahati, Assam, India. *

**Keywords:** Pediatric cancer, treatment abandonment, treatment refusal, survival

## Abstract

**Introduction:**

Refusal and abandonment of treatment is often considered as an important reason for poor survival of pediatric cancer patients in developing and underdeveloped countries. In this study we analyze the factors responsible for treatment abandonment and refusal in a Regional Cancer Centre (RCC) in North East India.

**Material and Methods:**

All histopathologically or cytologically confirmed cases of childhood cancer from below 15 years of age registered from 1st April, 2010 to 31st March, 2017 were included in this study. Parents or caregivers were interviewed thoroughly and a questionnaire was filled up for analysis of demographic and socio-economic factors. Modified Kuppuswamy scale was used to measure socioeconomic status.

**Results:**

Of 592 patients 161 (27.1%) abandoned therapy and 23 (3.9%) refused treatment. Factors associated with abandonment of treatment included: lower risk if residing in urban areas (Odds ratio [OR] = 0.8333, 95% CI 0.565-1.228; P=0.36) and higher risk with maternal education less than secondary school (OR = 1.357; 95%CI: 0.553-3.326; P=0.505). Low socioeconomic status and age >5yrs were also associated with abandonment of treatment. In a binary logistic regression analysis, male sex [Odds Ratio (OR) = 0.701; 95% CI 0.48-1.01; P=0.062] have lowest risk of abandoning treatment with trend to statistical significance.

**Conclusion:**

There is a need for proper definition of the problem of childhood cancer patients so that appropriate policy can be introduced to improve survival by improving treatment compliance.

## Introduction

With the advancement in diagnosis, treatment and supportive care in pediatric patients with cancer, there have been dramatic improvement in survival rates in developed nations, where 80% of the children with cancer achieve cure (Stiller et al., 2007; Mattesini et al., 2010). But picture is not so promising in developing and underdeveloped countries, which accounts for 70%-80% of the nearly 2,50,000 newly diagnosed childhood cancer cases each year; and, often less than 25% of them surviving (Kellie et al., 2008; Riberio et al., 2008). Refusal and abandonment of treatment is often considered as an important reason for inferior survival outcome in childhood cancers in low and middle income countries (Arora et al., 2007; Bonilla et al., 2009). In this study we have tried to analyze the factors responsible for treatment abandonment and refusal in a Regional Cancer Centre (RCC) in North East India since these were suspected to be major causes of treatment failure (Hazarika et al., 2014). We analyzed the association of demographic and disease related factors with increased risk of abandonment and refusal of treatment.

## Materials and Methods

Retrospective exploratory descriptive analysis was done after obtaining permission from Institutional Ethical Committee (IEC/BBCI-TMC/3187/2018)) of Dr. B Borooah Cancer Institute (BBCI), Guwahati, Assam, India, where hospital based cancer registration is going on actively since 2010. 

*Objectives of the study*

To analyze the association of demographic and disease related factors with increased risk of abandonment and refusal of treatment in patients with childhood cancer.

*Inclusion criteria*

All histopathologically or cytologically confirmed cases of childhood cancer below 15 years of age registered at our institution from 1^st^ April, 2010 to 31^st^ March, 2017 and had completed their diagnostic workup were included for the study. 

*Exclusion criteria*

Those with incomplete workup, previous diagnosis of cancer and who received any cancer directed treatment at other institute were excluded from the study. 

Diagnostic criteria were specific for different cancers as per institutional protocol. Compilation of the data was started from 1^st^ April 2018 and analysis was done on July 2018.

Gender, age, maternal education and socioeconomic status were included for measures. Demographic and socioeconomic factors including age, gender, occupation, family income and education were retrieved from Hospital based cancer registry, which was recorded at the time of hospital registration. Age groups were divided between age ≤5 yrs and >5 yrs. Place of residence was grouped into rural and urban. Maternal education was grouped into more than secondary school and less than secondary school education. Socioeconomic status was calculated by modified Kuppuswamy scale (Annexure 1) and divided into two groups one with total score >10 (middle and upper class) and another with score ≤10 (upper lower and lower class). Parents or caregivers were interviewed thoroughly telephonically and a questionnaire was filled up for causes of treatment abandonment analysis. On the basis of medical record review from patients, treatment outcome for each patient was categorized as: (1) completed or presently undergoing treatment; (2) refused treatment; (3) abandoned treatment/loss to follow up; or (4) death from any cause. 

Abandonment of treatment was defined as the termination of care by the parent/caregiver and/or not presenting for scheduled treatment for four weeks or more from the scheduled date of treatment at the time of data record in line with International Society of Pediatric Oncology (SIOP) recommendation (Mostert et al., 2011). If a child returned for treatment after 4 weeks, he/she was considered in treatment abandonment group for analysis. Refusal of treatment was defined as no initiation of treatment after the complete diagnosis of cancer. 

*Statistical Analyses*

Data management and analysis was done using SPSS version 19. A descriptive analysis for each factor was done. Difference in socio-demographic and clinical characteristics between treatment abandonment and treatment non abandonment were analyzed using χ^2^ tests. To verify factors that affected treatment abandonment, a binary logistic regression analysis was done. Two tailed p-values less than 0.05 were considered statistically significant at 95% confidence interval.

## Results

The median age at presentation was 7 years and mean age was 7.4 years (SD±4.8). The male: female ratio was 1.5: 1 in entire population. In our observation, out of 592 patients, 161 (27.1%) patients abandoned therapy and 23 (3.9%) patients refused treatment. Seventy two (31.2%) of 231 female patients abandoned treatment, whereas, 89 (24.7%) of 361 male patients abandoned treatment (Table 2), suggesting that more female patients tended to abandoned therapy. 

Other factors associated with abandonment of treatment included: lower risk in patients who were residing in urban areas versus those residing in rural areas [Odds ratio, (OR) = 0.8333; 95% CI: 0.565-1.228; P=0.36]; and higher risk in patients with maternal education less than secondary school versus those with maternal education more than secondary school education (OR = 1.357; 95% CI: 0.553-3.326; P= 0.505). Children coming from low socioeconomic status and age >5yrs were also associated with abandonment of treatment (Table 2). In a binary logistic regression analysis including gender, age, residence, socioeconomic status and maternal education, the associations with male sex (OR =0.701; 95%CI 0.48-1.01; P=0.062) had lowest risk of abandoning treatment with trend to statistical significance (Table 2). 

**Table 1:** Demographic and Treatment Characteristics

Variables	N=592 (%)	Median (Mean± SD)
Sex		
Male	361 (60.9)	
Female	231 (39.1)	
Age at diagnosis (in years)	-	7 (7.4±4.8)
Age (in years)		
>5 yrs	331 (55.9)	
≤5 yrs	261 (44.1)	
Maternal education		
More than secondary school	34 (5.7)	
Less than secondary school	551 (93.1)	
Unknown	7 (1.2)	
Residence		
Rural	385 (65)	
Urban	207 (35)	
Modified Kuppuswamy Scale score		
Upper lower and lower class (score ≤10)	467 (78.9)	
Upper and middle class (score >10)	125 (21.1)	
Treatment abandonment	161 (27.1)	
Treatment refused	23 (3.9)	

**Table 2:** Binary Logistic Regression Analysis of Biological and Socioeconomic Variables as Predictors for Treatment Abandonment

Variables	Treatment abandonmentn (%)	Non treatment abandonment n (%)	Odds ratio (OR)	95% CI	P value
Sex					
Female	72 (44.7)	159 (36.9)	1	0.483-1.018	0.062
Male	89 (55.3)	272 (63.1)	0.701		
Age at diagnosis (in years)					
>5 yrs	95 (59)	236 (54.8)	1	0.576-1.220	0.358
<5 yrs	66 (41)	195 (45.2)	0.839		
Maternal education					
More than secondary school	7 (4.5)	27 (6.3)	1		
Less than secondary school	150 (95.5)	401 (93.7)	1.357		
Residence					
Rural	100 (62.1)	285 (66.1)	1	0.565-1.228	0.356
Urban	61 (37.9)	146 (33.9)	0.8333		
Modified Kuppuswamy scale score					
Upper lower and lower class (score ≤10)	130 (80.7)	337 (78.2)	1	0.544-1.434	0.616
Upper and middle class (score >10)	31 ( 19.3)	94 (21.8)	0.883		

**Table 3:** Reasons of Treatment Abandonment

n=161 (%)	
Poor financial condition	46 (28.6%)
Progressive disease	36 (22.4%)
Long distance to treatment centre	20 (12.4%)
Unknown	48 (29.8%)
Toxicity	8 (4.9%)
Went to other centre	3 (1.9%)

**Table 4:** Reasons of Treatment Refusal

n=23(%)	
Long distance to treatment centre	11 (47.8%)
Poor financial condition	6 (26.1%)
Unknown	6 (26.1%)

In our observation, 46 (28.6%) patients abandoned treatment due to poor financial condition, 36 (22.4%) due to progressive disease and 20 (12.4%) due to long travelling distance. In our study, only 8 (4.9%) patients abandoned therapy due to treatment related toxicity, whereas, 3 (1.9%) patients went to other centers (Table 3). In 48 (29.8%) patients reason for treatment abandonment remains unknown (these includes patients who were not traceable and those who refused to participate in the study). 

The reasons for treatment refusal in our study were found to be long travelling distance from the area of residence to treatment center in 11 (47.8%) patients and poor financial status in 6 (26.1%) patients (table 4).

## Discussion

In our study, 27.1% of the patients abandoned treatment, which is consistent with other studies in developing countries where treatment abandonment were around 25–50% (Metzger et al., 2003; Meremikwu et al., 2005; Moster et al., 2006; De Boer et al., 2009; Mostert et al., 2010; Sitaresmi et al., 2010; Moster et al., 2012).

We did not find any statistically significant association of age group, residence, maternal education to affect treatment abandonment. Though majority of our patients belonged to lower socioeconomic class, this also did not appear to influence the treatment abandonment. However, we observe a trend for higher abandonment rate in female child which may be due gender inequality in Indian society favoring male child. 

Various reasons for abandonment of treatment are observed in different studies, most common being financial constraints and lack of parental education (Meremikwu et al., 2005; Arora et al., 2007; Jeremy et al., 2014). Monthly income of the family has also been shown to be significantly related to abandonment rate (Yadav et al., 2007; Bonilla et al., 2009). In our study 80.7% of the patients who abandoned treatment, the parents belonged to lower and upper lower socio-economic status by modified Kuppuswamy scale (Annexure: 1) (Kumar et al., 2007). We also observed more treatment abandonment if the mother had less than secondary school education. Similar finding were seen by Sachdeva et al., (2005) who reported that parents of children who abandoned treatment were found to have limited education and economic means.

Cost of transport and distance from treatment centre also contributes to treatment abandonment (Bonilla et al., 2009; Arora et al., 2010; Sitaresmi et al., 2010). Long distance from treatment centre was amongst the commonest reasons for treatment abandonment and also treatment refusal in our study. This may be because of many factors, including, poor connectivity of road and public transport due to difficult geographical location and the amount of time and money required to travel to the treatment center. Our hospital is the only tertiary cancer care centre for pediatric oncology in entire North-East India, where majority of the families seeking treatment had to travel more than 100 km distance. Opening satellite center, providing transportation cost with free lodging facility are few of the measures that can be done to reduce treatment abandonment and refusal. In Brazil, abandonment of treatment was nearly eliminated over a period of 20 years by providing lodging, food, and transportation assistance to patients with acute lymphoblastic leukemia (ALL) (Howard et al., 2004).

We observed that in patients belonging to rural areas treatment abandonment were higher (62%) as compared to patients coming from urban area, which may be due to long distance to travel or more frequent use of alternative mode of therapy. An Indian study by Kumar et al., (2013) in Retinoblastoma patients also showed more treatment abandonment in rural areas as seen in our study.

Age has been variably reported as being associated with treatment abandonment. In our study age more than 5 years was associated with more treatment abandonment. In contrast, Metzger et al., (2003) reported an association of abandonment with age less than 4.5 years in a cohort of children with ALL. However Arora et al., (2010) did not find any such association in cohort of patients from India. 

The percentage of male seeking healthcare was higher as compared to female in our study. When we analyzed gender as a separate factor, there was more treatment abandonment in female child. Similarly in a follow-up survey from Northern India, 28% of parents reported that the patient being a female influenced their decision for treatment abandonment (Arora et al., 2010).

Twenty three (3.9%) patients refused treatment in our study. This is comparatively less as compare to another study from a tertiary healthcare establishment in India, in which out of 762 children with ALL, 30% refused treatment (Kulkarni et al., 2009). This difference could be attributed to different disease population between these two studies. Refusal of treatment initiation is also a major cause of poor survival in pediatric cancer patients. In a study from Indonesia, it was seen that, after introduction of a parental education program to increase the access to information about leukemia for ALL patients, treatment refusal decreased from 14 to 2%, with improvement in survival (Mostert et al., 2010). Hence, improving systematic health education program with improvement of diagnostic evaluation facilities could have direct impact on reducing refusal rate.

Our study being retrospective one has got various limitations. As it was done after several years of diagnosis, we were unable to contact nearly one third families due to missing or outdated contact details in medical records and some declined to participate. Another limitation of this study includes the reliance on self-reported measures and recall bias. Other confounding factors like faith, cultural issues and communication between parents and health care provider may affect decision of treatment abandonment. Another drawback was to indentify best indicator for socioeconomic status as social transformation and fast-growing economy have rendered these scales ineffective. Socioeconomic measure used in our study (modified Kuppuswamy scale) also has limitations as there is an overemphasis on income rather than educational and occupational factors.

Even with these limitations, it is apparent that treatment abandonment is a complex issue. Also factors affecting abandonment in one population may not be applicable to another population. As such it is necessary to identify these factors to implement measures that reduce treatment abandonment and refusal. Provision of free treatment, better transport facility, and providing facility for accommodation will reduce economic burden to parents or other caregivers. There should be proper communication regarding systematic health education between parents and heath care providers with psychosocial support for health beliefs and experiences. It is also vital to track patients who did not report on timely visits for better compliance. 

In conclusion, there is an urgent need for proper definition of the problem of childhood cancer patients to implement appropriate policy to improve adherence to treatment. This observation will throw light on the causes of refusal and abandonment of treatment which is the leading cause of treatment failure in pediatric cancer cases in the developing world. A properly planned prospective study will help in better understanding of the issues so as to improve treatment adherence and thereby improving outcome.
